# Supplementary material for: Non-Hermitian Dirac cones with valley-dependent lifetimes
Source: Nat Commun. 2025 Feb 14;16:1627. doi: 10.1038/s41467-025-56882-y (PMC11825705; doi:10.1038/s41467-025-56882-y)
Supplement: Supplementary file 1 — Supplementary Information [file 41467_2025_56882_MOESM1_ESM.pdf]

Supplementary Information for
"Non-Hermitian Dirac cones with valley-dependent lifetimes"

Xinrong Xie[1,2,3,4,*], Fei Ma[1,2,3,4,*], W.B. Rui[5,6], Zhaozhen Dong[1,2,3,4], Yulin Du[1,2,3,4], Wentao Xie[7], Y.X. Zhao[5,6], Hongsheng Chen[1,2,3,4], Fei Gao[1,2,3,4,†], and Haoran Xue[7,‡],

[1]*State Key Laboratory of Extreme Photonics and Instrumentation, International Joint Innovation Center, The Electromagnetics Academy at Zhejiang University, Zhejiang University, Haining 314400, China*

[2]*ZJU-Hangzhou Global Scientific and Technological Innovation Center, Zhejiang University, Hangzhou 310027, China*

[3]*Key Lab. of Advanced Micro/Nano Electronic Devices & Smart Systems of Zhejiang, Jinhua Institute of Zhejiang University, Zhejiang University, Jinhua 321099, China*

[4]*Shaoxing Institute of Zhejiang University, Zhejiang University, Shaoxing 312000, China*

[5]*Department of Physics and HKU-UCAS Joint Institute for Theoretical and Computational Physics at Hong Kong, The University of Hong Kong, Pokfulam Road, Hong Kong, China*

[6]*HK Institute of Quantum Science & Technology, The University of Hong Kong, Pokfulam Road, Hong Kong, China*

[7]*Department of Physics, The Chinese University of Hong Kong, Shatin, Hong Kong SAR, China*

† ‡ Correspondence to: gaofeizju@zju.edu.cn (F. G.); haoranxue@cuhk.edu.hk (H. X.)

* These authors contributed equally: Xinrong Xie, Fei Ma.

Table of Contents:

S1. Calculated bulk dispersions with the tight-binding model.

S2. Implementation of the nonreciprocal capacitance

S3. Theoretical model of the designed electric circuits

S4. Valley-dependent Chern number

S5. Calculated interface dispersions with the tight-binding model.

S6. Point-gap topology of the valley-kink states

S7. Experimental measurement setup.

S8. Realizing non-Hermitian Dirac cones in other platforms.

S1. CALCULATED BULK DISPERSIONS WITH THE TIGHT-BINDING MODEL.

Figure S1 shows the calculated dispersions for the non-Hermitian Dirac cones obtained from the lattice Hamiltonian (Eq. (1) in the main text) and the effective Hamiltonian (Eq. (2) in the main text). These results are consistent for both the massless (Fig. S1a and S1b) and massive (Fig. S1c and S1d) cases.

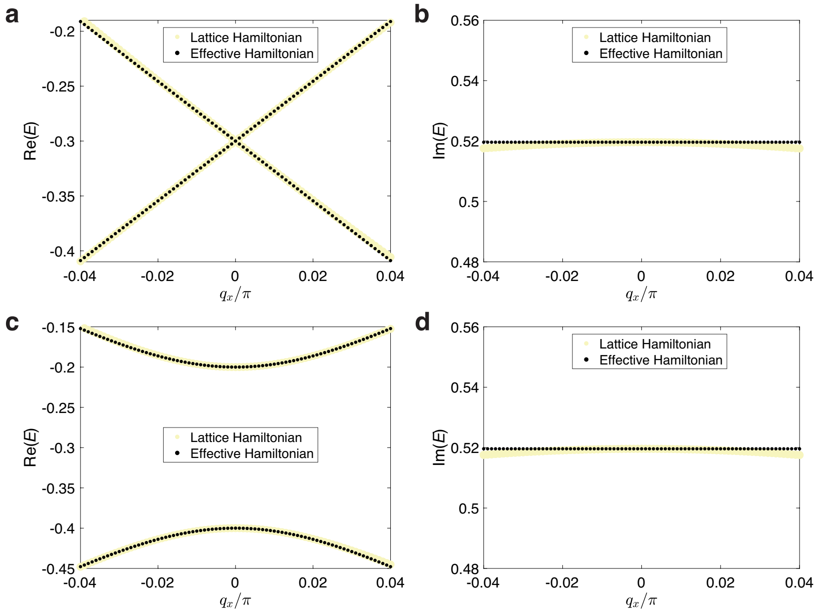

FIG. S1. Dispersions for the non-Hermitian Dirac cones obtained from the lattice Hamiltonian (yellow dots) and the effective Hamiltonian (black dots) for $m = 0$ ((**a**) and (**b**)) and $m = 0.1$ ((**c**) and (**d**)). Other parameters are $t = 1$, $t_2 = 0.1$, and $\delta = 0.1$.

S2. IMPLEMENTATION OF THE NONRECIPROCAL CAPACITANCE.

In this section, we clarify the implementation of the nonreciprocal capacitance. Figure S2 shows the circuit diagram of the nonreciprocal capacitance, realized by connecting a negative impedance converter with current inversion (INIC) in parallel with a capacitor C_1. The INIC (highlighted with the red dashed line) consists of an operational amplifier, which is an active circuit element, and some passive circuit elements, including two equivalent impedances Z_0 and a capacitor C_2. Based on the characteristics of the operational amplifier, we get the

following equation by carrying out Kirchhoff's law on the circuit node 1 and node 2

$$I_1 = -i\omega(C_1 - C_2)(V_1 - V_2) \tag{1}$$

$$I_2 = -i\omega(C_1 + C_2)(V_2 - V_1) \tag{2}$$

We can rewrite the relations between the currents and the node voltages in the matrix formalism as

$$\begin{pmatrix} I_1 \\ I_2 \end{pmatrix} = J \begin{pmatrix} V_1 \\ V_2 \end{pmatrix} = i\omega \begin{bmatrix} -(C_1 - C_2) & C_1 - C_2 \\ C_1 + C_2 & -(C_1 + C_2) \end{bmatrix} \begin{pmatrix} V_1 \\ V_2 \end{pmatrix} \tag{3}$$

where J is the admittance matrix of the circuit structure. It can be seen that the capacitance from left to right $(C_1 - C_2)$ is not equal to the capacitance from right to left $(C_1 + C_2)$, showing the nonreciprocity.

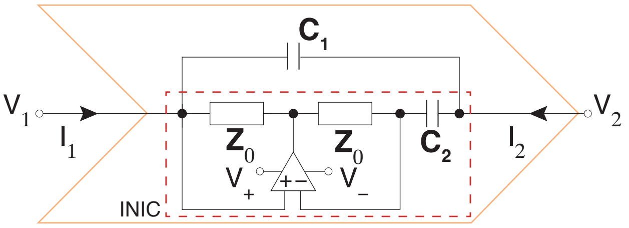

FIG. S**2**. Circuit diagram of the nonreciprocal capacitance. It is constructed by connecting the negative impedance converter with current inversion (INIC) in parallel with a capacitor C_1.

S3. THEORETICAL MODEL OF THE DESIGNED ELECTRIC CIRCUITS

According to Kirchhoff's law, the circuit model can be represented by the admittance matrix, also termed as a circuit Laplacian $J(\omega)$. The circuit Laplacian describes the voltage response $V(\omega)$ to an alternating-current input $I(\omega)$ according to $I(\omega) = (D(\omega) - E(\omega) + W(\omega))V(\omega) = J(\omega)V(\omega)$, where ω is the angular driving frequency. $D(\omega)$ and $W(\omega)$ are diagonal matrices containing the total conductances of each node to the other nodes and the ground, respectively. $E(\omega)$ is the adjacency matrix of the conductances.

As we can see in Fig. S3a, each unit cell possesses two nodes. In this case, the voltage at the node A/B is expressed as $V^r_{a/b}$, and the current is labeled as $I^r_{a/b}$. Additionally, each node

is connected with other sites through two kinds of coupling: nearest-neighbor coupling (C) and nonreciprocal next-nearest-neighbor coupling (nonreciprocal capacitance $C_1 \mp C_2$ along the arrow). Also, each node is grounded through an LC resonant circuit (L and $C_{a/b}$ for site A/B). Additionally, a resistor (R_s) is added from each node to the ground to guarantee the circuit stability. Using the Kirchhoff's current formula, we can write currents that flow into each node as

$$\begin{aligned} I_a^r &= i\omega C\left[(V_a^r - V_b^{r-a_2}) + (V_a^r - V_b^{r+a_3}) + (V_a^r - V_b^r)\right] \\ &+ i\omega(C_1 - C_2)\left[(V_a^r - V_a^{r+a_1}) + (V_a^r - V_a^{r+a_2}) + (V_a^r - V_a^{r+a_3}\right] \\ &+ i\omega(C_1 + C_2)\left[(V_a^r - V_a^{r-a_1}) + (V_a^r - V_a^{r-a_2}) + (V_a^r - V_a^{r-a_3}\right], \\ &+ (i\omega C_a + \frac{1}{i\omega L} - \frac{1}{R_s})V_a^r \end{aligned} \tag{4}$$

$$\begin{aligned} I_b^r &= i\omega C\left[(V_b^r - V_a^r) + (V_b^r - V_a^{r-a_3}) + (V_b^r - V_a^{r+a_2})\right] \\ &+ i\omega(C_1 - C_2)\left[(V_b^r - V_b^{r+a_1}) + (V_b^r - V_b^{r+a_2}) + (V_b^r - V_b^{r+a_3}\right] \\ &+ i\omega(C_1 + C_2)\left[(V_b^r - V_b^{r-a_1}) + (V_b^r - V_b^{r-a_2}) + (V_b^r - V_b^{r-a_3}\right]. \\ &+ (i\omega C_b + \frac{1}{i\omega L} - \frac{1}{R_s})V_b^r \end{aligned} \tag{5}$$

Then assuming there is no inputting current and using the Bloch theorem such as $V_b^{r+a_1} = V_b^r exp(i\boldsymbol{k}\cdot\boldsymbol{a_1})$, we obtain the eigenequation of the designed circuit

$$H^{\mathrm{c}}(\mathbf{k})\mathbf{V} = (\frac{1}{\omega^2 L} - i\frac{1}{\omega R_s})\mathbf{V}, \tag{6}$$

where $\mathbf{V} = [V_a, V_b]^{\mathrm{T}}$ are the node voltages on the two sublattices and ω is the angular frequency. $H^{\mathrm{c}}(\mathbf{k}) = \sum_{i=0,1,2,3} h_i^{\mathrm{c}}(\mathbf{k})\sigma_i$ is the Hamiltonian of the circuit, with $h_0^{\mathrm{c}} = \sum_{i=1,2,3} -2[C_1\cos(\mathbf{k}\cdot\mathbf{a}_i) - iC_2\sin(\mathbf{k}\cdot\mathbf{a}_i)] + 3C + 6C_1 + (C_a + C_b)/2$, $h_1^{\mathrm{c}} = -C[1 + \cos(\mathbf{k}\cdot\mathbf{a}_2) + \cos(\mathbf{k}\cdot\mathbf{a}_3)]$, $h_2^{\mathrm{c}} = -C[\sin(\mathbf{k}\cdot\mathbf{a}_2) - \sin(\mathbf{k}\cdot\mathbf{a}_3)]$, and $h_3^{\mathrm{c}} = (C_a - C_b)/2$. Here $\mathbf{k} = (k_x, k_y)$ is the wavevector, $\mathbf{a}_1 = (1,0)^{\mathrm{T}}$, $\mathbf{a}_2 = (-1/2, \sqrt{3}/2)^{\mathrm{T}}$ and $\mathbf{a}_3 = (-1/2, -\sqrt{3}/2)^{\mathrm{T}}$ are lattice vectors, σ_0 is the identity matrix, $\sigma_{1,2,3}$ are the Pauli matrices.

H^{c} has a similar form to the tight-binding Hamiltonian (see Eq. 1 in the main text), except that H^{c} contains a global offset ($3C + 6C_1$), which only globally shifts the eigenvalues along the real axis but has no influences on the eigenstates [1].

For a finite circuit, additional circuit elements are added to the nodes at the boundary (nodes denoted with green circles in Fig. S3b), including suitable capacitors for the same diagonal elements of the circuit Laplacian and absorbing resistors $R_a = 30\ \Omega$ to eliminate the

reflections. Taking the node marked with a black arrow as an example, additional ground capacitance $C + 4C_1$ is added, making it similar to the nodes inside the bulk with the same offset $3C + 6C_1$.

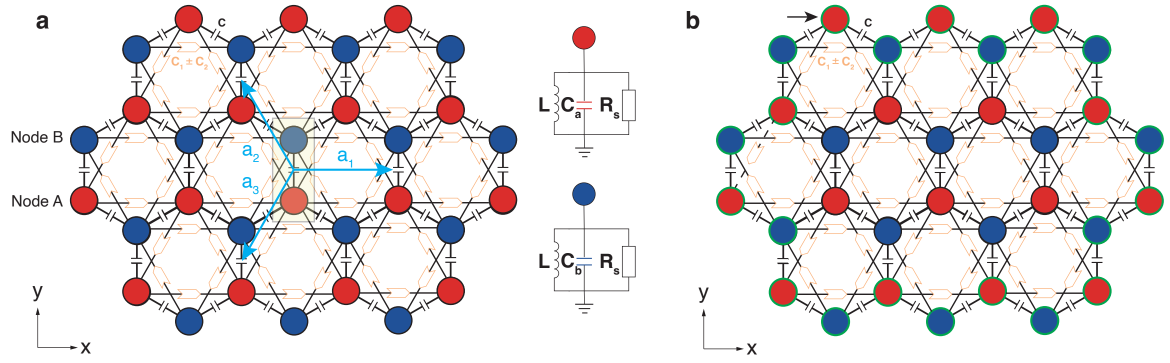

FIG. S3. Schematic of the non-Hermitian graphene circuit lattice with periodic **a**, and open **b** boundary conditions. **a**, A unit cell is highlighted with a rectangular box. $\boldsymbol{a_1}$, $\boldsymbol{a_2}$, and $\boldsymbol{a_3}$ denote the lattice vectors. **b**, Sites denoted with green circles require additional capacitance to achieve the same diagonal elements in the circuit Laplacian and absorbing resistors $R_a = 30\ \Omega$ to eliminate the reflections.

S4. VALLEY-DEPENDENT CHERN NUMBER

The Hamiltonian (Eq. (2) in the main text) produces a nontrivial valley-dependent Berry curvature with a distribution sharply centered at the two valleys. The normalized eigenvector of the lower band is

$$u(q_x, q_y) = \frac{1}{\sqrt{1 + \frac{q_x^2+q_y^2}{\left(\sqrt{m^2+q_x^2+q_y^2}+m\right)^2}}} \begin{pmatrix} -\frac{q_x+iq_y}{\sqrt{m^2+q_x^2+q_y^2}+m} \\ 1 \end{pmatrix}. \quad (7)$$

The Berry connection can be calculated according to the definition

$$\mathbf{A}(q_x, q_y) = -i\langle u(q_x, q_y)|\nabla_k|u(q_x, q_y)\rangle = \frac{1 + \frac{m}{\sqrt{m^2+q_x^2+q_y^2}}}{2\left(q_x^2 + q_y^2\right)} \left(q_y\mathbf{x} - q_x\mathbf{y}\right), \quad (8)$$

where $\nabla_k = \left(\frac{\partial}{\partial q_x}, \frac{\partial}{\partial q_y}\right)$. Therefore, we get the results of the Berry curvature

$$\Omega_K = \frac{\partial A_x}{\partial q_y} - \frac{\partial A_y}{\partial q_x} = \frac{m}{2\left(m^2 + q_x^2 + q_y^2\right)^{\frac{3}{2}}}. \quad (9)$$

The calculated Berry curvature of positive (negative) mass terms is shown in Fig. S4A (Fig. S4B). According to the definition of the valley Chern number $C_K = \frac{1}{2\pi}\int_{\mathrm{HBZ}} q_x q_y \Omega_K$, the valley Chern number $C_K = \mathrm{sgn(m)}/2$.

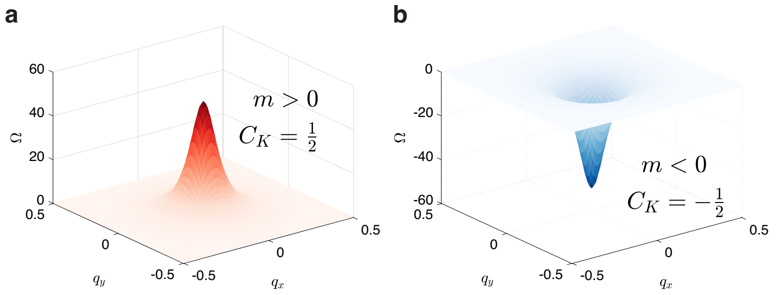

FIG. S4. Berry curvature around the K valley. **a**, mass term $m > 0$. **b**, mass term $m < 0$.

S5. CALCULATED INTERFACE DISPERSIONS WITH THE TIGHT-BINDING MODEL.

It is known that an interface between two domains with opposite Dirac masses can support valley gapless valley kink states, which are protected by the valley Chern numbers. For the zigzag interface constructed by a lattice with $m = -0.5$ at the upper domain and a lattice with $m = 0.5$ at the lower domain, the valley kink states are observed with additional valley-dependent imaginary eigenvalues (Fig. S5a), which illustrate the non-Hermitian boundary physics originating from the bulk.

We further demonstrate that the non-Hermitian valley kink states are more robust than their Hermitian counterparts. To see this , we consider the armchair interface where the projections of the two valleys overlap. Figure S5b plots the evolution of the eigenvalues of the kink states at $k_y = 0$ (where the kink states cross each other) as a function of the dimensionless non-Hermitian parameter δ/t_2. As δ/t_2 increases, the gap size decreases and eventually becomes zero after a parity-time phase transition. Figure S5c and S5d show the calculated dispersions before and after the phase transition, respectively. It can be clearly seen that the edge states after the phase transition are gapless and have contrasting imaginary eigenvalues, similar to the zigzag case. These results are consistent with the calculations from the circuit model in the main text.

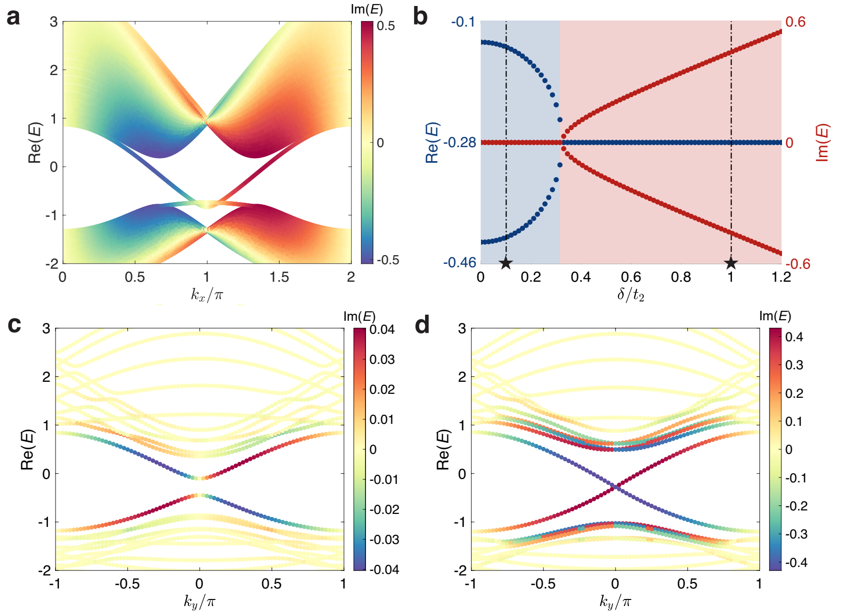

FIG. S5. Calculated interface dispersions with the tight-binding model. **a**, Calculated dispersion for a heterostructure with a zigzag interface constructed by a lattice with $m = -0.5$ at the upper domain and a lattice with $m = 0.5$ at the lower domain. Other parameters are $t = 1$, $t_2 = 0.1$, and $\delta = 0.1$. **b**, Plots of the real (blue dots) and imaginary (red dots) parts of the valley kink states' eigenfrequencies at $k_y = 0$ of a heterostructure with an armchair interface against δ/t_2. The blue and red regions denote the parity-time symmetric and parity-time symmetry-broken phases, respectively. Other parameters are $m = 0.5$, $t = 1$, and $t_2 = 0.1$. **c-d**, Calculated dispersions for the two cases denoted by the black states in (**a**) ($\delta/t_2 = 0.1$ for (**c**) and $\delta/t_2 = 1$ for (**d**)). The colors denote the imaginary parts of eigenvalues.

S6. POINT-GAP TOPOLOGY OF THE VALLEY-KINK STATES

From the non-Hermitian topology point of view, the non-Hermitian valley interface mimics the classic Hatano-Nelson model with a non-trivial point-gap topology [2]. When periodic boundary conditions are imposed, the non-Hermitian zigzag valley interface exhibits a set of delocalized states with pair-wise complex eigenvalues, forming a point gap in the spectrum as illustrated by black dots in Fig. S6a. The topological properties of the point gap are characterized by the winding number [3]:

$$w = -i \int_{\mathrm{BZ}} \frac{dk}{2\pi} \mathrm{Tr}[Q(k)], \tag{10}$$

with $Q(k) = [H(k) - E]^{-1}\partial_k[H(k) - E]$, E is any complex value in the point gap, and $\mathrm{BZ} = [-\pi, \pi]$ represents the Brillouin zone. By choosing $E = 0.2$, we find that the point gap is topologically non-trivial, characterized by a nonzero winding number of $w = 1$.

In the case of open boundary conditions, some purely real eigenvalues emerge in the original point-gap region, as illustrated in Fig. S6a. The corresponding eigenstates exhibit the non-Hermitian skin effects, whereby the energy distribution along the interface accumulates at the right ends. Fig. S6b displays one of the localized eigenstates, corresponding to the eigenvalue denoted with a black arrow in Fig. S6a. Note that our experiments do not observe the skin modes since absorbing boundaries are implemented in our samples.

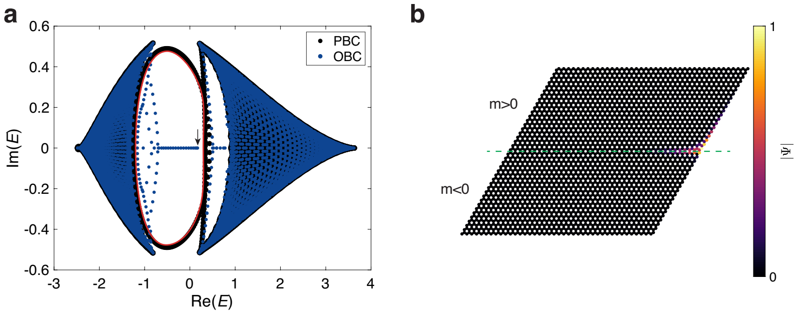

FIG. S6. Point-gap topology of the valley-kink states **a**, Eigenvalues of the non-Hermitian zigzag valley interface when periodic (black dots), or open (blue dots) boundary conditions are applied. The nearest-neighbor coupling coefficient is $t = 1$, the NNN coupling coefficient is $t_2 \pm \delta = 0.1 \pm 0.1$, and the mass term is $m = \pm 0.5$. **b**, The eigenstate distribution corresponding to the eigenvalue highlighted with a black arrow in (**a**). The greed dashed line denotes the zigzag interface.

S7. EXPERIMENTAL MEASUREMENT SETUP.

The experiment measurement setup is shown in Fig. S7. The voltage is measured by a two-port network vector analyzer (KEYSIGHT N9927A). We place port 1 at the excitation node and use port 2 to measure the voltage responses $U_{x,y}$ of all the other nodes. By applying the Fourier transformation to $U_{x,y}$. we obtain the dispersion diagrams. The DC supplies (KORAD KA3005DS) are used to provide ±5 V DC voltages for the optional amplifiers. In the measurements, the output power is -3 dBm, the frequency sweep size is 50 Hz, and the intermediate frequency bandwidth (IFBW) is 3 kHz, which all help to reduce measurement errors.

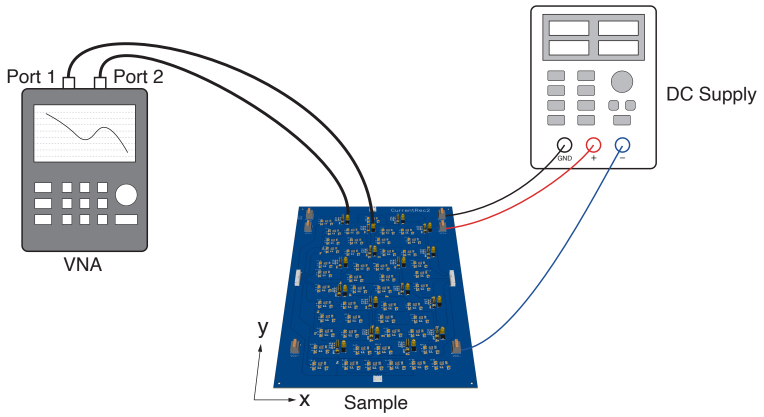

FIG. S7. Experimental setup for the voltage response measurement.

S8. REALIZING NON-HERMITIAN DIRAC CONES IN OTHER PLATFORMS.

In addition to the electric circuits with INIC, there are other ways to realize the nonreciprocal couplings experimentally in electronic, photonic, acoustic, and mechanical systems. In this section, we validate the feasibility of realizing non-Hermitian Dirac cones in acoustic and mechanical systems with existing schemes of nonreciprocal couplings, by presenting numerically calculated dispersions using realistic parameters.

A. Acoustic implementation of non-Hermitian Dirac cones

In acoustics, we follow the scheme proposed in Ref. [4, 5] to realize the non-Hermitian Dirac cone model in this work. As schematically shown in Fig. S8a, the structure consists of identical air cavities (blue and red circles) with a fundamental dipole resonance frequency f_0, and an intrinsic loss γ_0. The coupling methods are shown in Fig. S8b. The reciprocal nearest-neighbor coupling t is realized by connecting air cavities with narrow tubes. The nonreciprocal coupling $\widetilde{\kappa} = \rho e^{i\theta}$ with tunable amplitude ρ and phase θ is realized with a unidirectional coupler, which consists of a microphone, an amplifier, a phase shifter, and a loudspeaker. The corresponding Bloch Hamiltonian reads

$$H^{\mathrm{a}}(\mathbf{k}) = \sum_{i=0}^{3} h_i^{\mathrm{a}}(\mathbf{k})\sigma_i, \tag{11}$$

where $h_0^{\mathrm{a}} = \sum_{i=1,2,3} f_0 - i\gamma + \widetilde{\kappa}[\cos(\mathbf{k}\cdot\mathbf{a}_i) - i\sin(\mathbf{k}\cdot\mathbf{a}_i)]$, $h_1^{\mathrm{a}} = t[1+\cos(\mathbf{k}\cdot\mathbf{a}_2)+\cos(\mathbf{k}\cdot\mathbf{a}_3)]$, $h_2^{\mathrm{a}} = t[\sin(\mathbf{k}\cdot\mathbf{a}_2) - \sin(\mathbf{k}\cdot\mathbf{a}_3)]$, $h_3^{\mathrm{a}} = 0$, σ_0 is the identity matrix, and $\sigma_{1,2,3}$ are the Pauli

matrices. Here $\mathbf{k} = (k_x, k_y)$ is the wavevector, $\mathbf{a}_1 = (1,0)^{\mathrm{T}}$, $\mathbf{a}_2 = (-1/2, \sqrt{3}/2)^{\mathrm{T}}$ and $\mathbf{a}_3 = (-1/2, -\sqrt{3}/2)^{\mathrm{T}}$ are lattice vectors.

Here we use the parameters that have been realized in Ref. [5], i.e., $f_0 = 5178$ Hz, $\gamma_0 = 27.5$ Hz, $t = 44.4$ Hz, $\rho = 6.44$ Hz, and $\theta = 0$. Figure S8c shows the calculated dispersion. Two Dirac cones with different imaginary eigenfrequencies can be clearly seen at the K and K′ valley, similar to the circuit case.

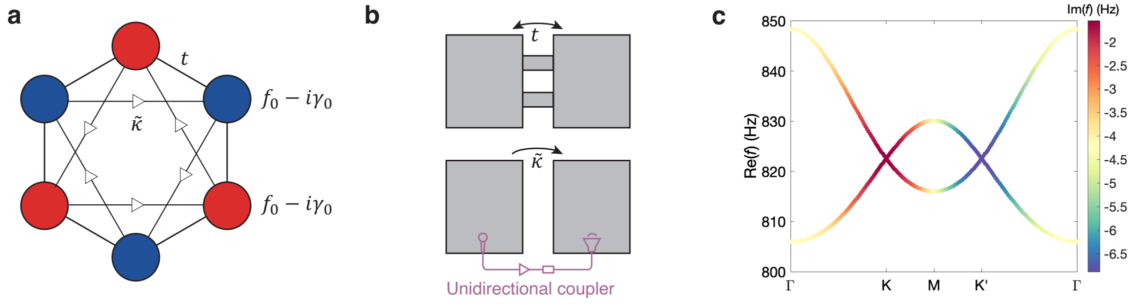

FIG. S8. Acoustic implementation of non-Hermitian Dirac cones. **a**, The schematic diagram of the designed acoustic structure realizing the hexagonal cell. Identical air cavities (red and blue circles) have a fundamental dipole resonance frequency f_0 and an intrinsic loss γ_0. **b**, Coupling mechanisms. The reciprocal nearest-neighbor coupling t is realized by connecting air cavities with narrow tubes. The nonreciprocal coupling $\widetilde{\kappa}$ is realized with a unidirectional coupler, which consists of a microphone, an amplifier, a phase shifter, and a loudspeaker. **c**, Calculated bulk dispersion of the acoustic lattice with parameters $f_0 = 5178$ Hz, $\gamma_0 = 27.5$ Hz, $t = 44.4$ Hz, and $\widetilde{\kappa} = 6.44$ Hz. The colors denote the imaginary part of the eigenfrequencies.

B. Mechanical implementation of non-Hermitian Dirac cones

In mechanics, we follow the scheme proposed in Ref. [6, 7] to realize the non-Hermitian Dirac cones. The schematic diagram is shown in Fig. S9a. The building block is a rotational oscillator consisting of a brushless motor and a rotational arm connected to two anchor points via two tensioned springs with the on-site resonant frequency f_0 and the on-site loss γ. The coupling methods are shown in Fig. S9b. The oscillators are connected by tensioned springs to produce the reciprocal nearest-neighbor coupling t. The nonreciprocal coupling $t_2 \pm \delta$ is realized by programmed external actuation of the motors. The corresponding Bloch

Hamiltonian reads

$$H^{\mathrm{m}}(\mathbf{k}) = \sum_{i=0}^{3} h_i^{\mathrm{m}}(\mathbf{k})\sigma_i, \tag{12}$$

where $h_0^{\mathrm{tb}} = \sum_{i=1,2,3} f_0 - i\gamma + 2[t_2 \cos(\mathbf{k}\cdot\mathbf{a}_i) - i\delta \sin(\mathbf{k}\cdot\mathbf{a}_i)]$, $h_1^{\mathrm{m}} = t[1 + \cos(\mathbf{k}\cdot\mathbf{a}_2) + \cos(\mathbf{k}\cdot\mathbf{a}_3)]$, $h_2^{\mathrm{m}} = t[\sin(\mathbf{k}\cdot\mathbf{a}_2) - \sin(\mathbf{k}\cdot\mathbf{a}_3)]$, $h_3^{\mathrm{m}} = m$, σ_0 is the identity matrix, and $\sigma_{1,2,3}$ are the Pauli matrices. Here $\mathbf{k} = (k_x, k_y)$ is the wavevector, $\mathbf{a}_1 = (1,0)^{\mathrm{T}}$, $\mathbf{a}_2 = (-1/2, \sqrt{3}/2)^{\mathrm{T}}$ and $\mathbf{a}_3 = (-1/2, -\sqrt{3}/2)^{\mathrm{T}}$ are lattice vectors.

Here we use the parameters that have been realized in Ref. [7], i.e., $f_0 = 54.67$ Hz, $\gamma = 1.82$ Hz, $t = -2.33$ Hz, $t_2 = -0.38$ Hz, and $\delta = -0.1$ Hz. Figure. S9c shows the calculated dispersion. Two Dirac cones with different imaginary eigenfrequencies can be clearly seen at the K and K′ valley, similar to the circuit case.

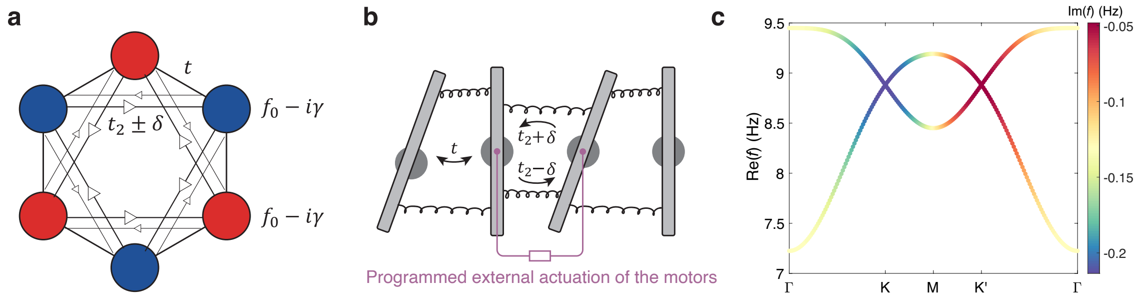

FIG. S9. Mechanical implementation of non-Hermitian Dirac cones. **a**, The schematic diagram of the designed mechanical structure realizing the hexagonal unit cell. Identical rotational oscillators (red and blue circles) have the on-site resonant frequency f_0 and an intrinsic loss γ. **b**, Coupling mechanisms. The reciprocal nearest-neighbor coupling t is realized by connecting rotational oscillators with tensioned springs. The nonreciprocal coupling $t_2 \pm \delta$ is realized by programmed external actuation of the motors. **c**, Calculated bulk dispersion of the acoustic lattice with parameters $f_0 = 54.67$ Hz, $\gamma = 1.82$ Hz, $t = -2.33$ Hz, $t_2 = -0.38$ Hz, and $\delta = -0.1$ Hz. The colors denote the imaginary part of the eigenfrequencies.

[1] T. Helbig, T. Hofmann, S. Imhof, M. Abdelghany, T. Kiessling, L. W. Molenkamp, C. H. Lee, A. Szameit, M. Greiter, and R. Thomale, Generalized bulk–boundary correspondence in non-Hermitian topolectrical circuits, Nat. Phys. **16**, 747 (2020).

[2] N. Hatano and D. R. Nelson, Localization transitions in non-Hermitian quantum mechanics, Phys. Rev. Lett. **77**, 570 (1996).

[3] N. Okuma, K. Kawabata, K. Shiozaki, and M. Sato, Topological origin of non-Hermitian skin effects, Phys. Rev. Lett. **124**, 086801 (2020).

[4] L. Zhang, Y. Yang, Y. Ge, Y.-J. Guan, Q. Chen, Q. Yan, F. Chen, R. Xi, Y. Li, D. Jia, S.-Q. Yuan, H.-X. Sun, H. Chen, and B. Zhang, Acoustic non-Hermitian skin effect from twisted winding topology, Nat. Commun. **12**, 6297 (2021).

[5] Q. Zhang, Y. Li, H. Sun, X. Liu, L. Zhao, X. Feng, X. Fan, and C. Qiu, Observation of acoustic non-Hermitian Bloch braids and associated topological phase transitions, Phys. Rev. Lett. **130**, 017201 (2023).

[6] A. Ghatak, M. Brandenbourger, J. V. Wezel, and C. Coulais, Observation of non-Hermitian topology and its bulk–edge correspondence in an active mechanical metamaterial, Proc. Natl. Acad. Sci. U.S.A. **117**, 29561 (2020).

[7] W. Wang, X. Wang, and G. Ma, Non-Hermitian morphing of topological modes, Nature **608**, 50 (2022).
